# Mesenchymal stem cells internalize *Mycobacterium tuberculosis* through scavenger receptors and restrict bacterial growth through autophagy

**DOI:** 10.1038/s41598-017-15290-z

**Published:** 2017-11-08

**Authors:** Arshad Khan, Lovepreet Mann, Ramesha Papanna, Mi-Ae Lyu, Christopher R. Singh, Scott Olson, N. Tony Eissa, Jeffrey Cirillo, Gobardhan Das, Robert L. Hunter, Chinnaswamy Jagannath

**Affiliations:** 1 Dept. of Pathology and Laboratory Medicine, University of Texas Health Sciences Center, Houston, TX 77030 USA; 2 Dept. of Obstetrics, Gynecology and Reproductive Sciences, UTHSC-, Houston, USA; 3 Dept. of Pediatric Surgery, UTHSC-, Houston, USA; 4 Dept. of Pulmonary Medicine, Baylor college of Medicine, Houston, TX USA; 5 Dept. of Microbial Pathogenesis and Immunology, Center for Airborne Pathogens Research and Imaging, Texas A&M Health Science Center, College of Medicine, Bryan, USA; 6 Center for Molecular Medicine, Jawaharlal Nehru University, New Delhi, India

## Abstract

Human mesenchymal stem cells (MSCs) express scavenger receptors that internalize lipids, including oxidized low-density lipoprotein (oxLDL). We report that MSCs phagocytose *Mycobacterium tuberculosis* (Mtb) through two types of scavenger receptors (SRs; MARCO and SR-B1), as blockade of the receptors with antibodies or siRNA knockdown decreased the uptake of Mtb. MSCs also expressed mannose receptor (MR) that was found to endocytose rhodamine-labeled mannosylated BSA (rMBSA), though the receptor was not involved in the uptake of Mtb. Dil-oxLDL and rMBSA taken up into MSC endosomes colocalized with Mtb phagosomes, thus suggesting that the latter were fusion competent. Phagocytosed Mtb did not replicate within MSCs, thus suggesting an intrinsic control of bacterial growth. Indeed, MSCs exhibited intrinsic autophagy, which was up-regulated after activation with rapamycin. SiRNA knockdown of autophagy initiator beclin-1 enhanced Mtb survival, whereas rapamycin-induced autophagy increased intracellular killing of Mtb. In addition, MSCs secreted nitric oxide after Mtb infection, and inhibition of NO by N(G)-monomethyl-L-arginine enhanced intracellular survival of Mtb. MSCs can be grown in large numbers *in vitro*, and autologous MSCs transfused into tuberculosis patients have been found to be safe and improve lung immunity. Thus, MSCs are novel phagocytic cells with a potential for immunotherapy in treating multidrug-resistant tuberculosis.

## Introduction

Tuberculosis is responsible for 2 million deaths each year and is a major cause of mortality from a single infectious disease worldwide^1^. According to the WHO, some parts of the former Soviet Union, show up to 28% of new tuberculosis cases as multidrug-resistant^2^. Today, drug-resistant tuberculosis is quite common in India and China, the two countries with the highest MDR-TB burdens (TBfacts.org). Tuberculosis is therefore a disease for which novel intervention strategies are required to supplement available drug regimens.

*Mycobacterium tuberculosis* (Mtb) parasitizes macrophages and has special evasion mechanisms that enable it to survive, multiply and spread infection. When internalized by macrophages, Mtb is located within an immature membrane-bound phagosome, which can fuse or mature into a lysosome^3^. In immunocompetent people, Th1 immunity against Mtb is generated through degradation of Mtb in lysosomes, thus producing peptides that are routed via MHC-II to CD4 T cells. These cells secrete Th1 cytokines, which in turn activate macrophages and increase the secretion of oxidants, which kill Mtb. In addition, Th1 cytokines such as IFN-γ enhance phagosome-lysosome fusion^4^. Secreted proteins (e.g., Ag85B, ESAT6, CFP10) of Mtb escape into the cytosol, and peptides cleaved by proteasomes are loaded through MHC-I and activate CD8 T cells. These cells secrete IFN-γ and are able to lyse Mtb-infected macrophages through perforin and granzyme. Whereas both CD4 and CD8 T cells are critical for anti-tuberculosis immunity, depletion of CD4 T cells is a major predisposing factor for lethal HIV-1 and tuberculosis coinfection^5,6^.

The primary evasion mechanism of Mtb is the inhibition of phagosome-lysosome (PL) fusion within macrophages, thus preventing killing and degradation of the pathogen^7^. Second, Mtb secretes powerful anti-oxidants that neutralize the reactive oxygen radicals^8^. Third, Mtb has a lipid-rich cell wall, which makes the bacterium resistant to multiple defensive mechanisms. Macrophages must be activated by cytokines such as IFN-γ and TNF-α for enhanced secretion of oxidants such as nitric oxide and superoxide, which kill Mtb. In addition, IFN-γ enhances PL fusion for efficient killing of Mtb^6,9^. Because Mtb has multiple evasion mechanisms, it can persist in a dormant state in macrophages, and consequently is difficult to eradicate. Thus, macrophages occupy a crucial position at the center of the classic lesion of human tuberculosis and are surrounded by a cuff of immune cells, such as DCs, T cells, neutrophils and eosinophils^10^.

Mesenchymal stem cells (MSCs) are a heterogeneous subset of stromal stem cells, which are readily isolated from many adult tissues^11–13^. They are multipotent cells that can differentiate into adipocytes, osteoblasts, chondrocytes and neuronal cells. Even though MSCs are not known to interact with microbial pathogens, they express certain Toll-like receptors (TLRs), NOD2 and RIG-I^14–16^. MSCs treated with lipopolysaccharide (LPS), which activates TLR-4, undergo induction of osteogenic differentiation, whereas dsRNA activates TLR-3 and enhances stem cell migration^17^. MSCs are thought to interact with various components of the immune system and to modulate immune responses by affecting the M1 and M2 phenotypes of macrophages and dendritic cells^18^. However, the cell biology of MSCs during infection has remained largely unclear^11,12^.

We have previously reported the novel observation that MSCs are present around tuberculosis granulomas in mice, and they suppress the T-lymphocyte responses of mice through the secretion of nitric oxide^19^. Human tuberculosis granulomas also contain infiltrating MSCs with acid-fast bacteria (AFB), and others have expanded on this observation^20,21^. Additionally, AFB and DNA from Mtb have been found within MSCs obtained from tuberculosis patients^20,21^. Interestingly, phase I clinical trials have shown that infusion of autologous MSCs is safe and improves lung function and immunity among patients with MDR/XDR tuberculosis. When administered with second-line drugs, stem cell therapy improves the clinical condition of these patients^22^.

Unlike other immune cells, MSCs can be grown *in vitro* in large numbers and infused back into patients. However, lack of mechanistic knowledge about their cell biology has been a major impediment to the optimization stem of cell therapy^11^. Herein, we describe a unique property of MSCs: that they function as novel phagocytic immune cells and exhibit intrinsic control of mycobacterial replication. MSCs were found to internalize Mtb through two types of scavenger receptors: MARCO (macrophage receptor with collagenous structure) and SR-B1 (a.k.a. CD36). We demonstrate that live Mtb does not multiply in MSCs, owing to their intrinsic autophagy, and rapamycin-induced autophagy enhances intracellular killing of the pathogen. In addition, MSCs secrete NO, which restricts the growth of Mtb. We therefore propose a novel phagocyte-like function of MSCs during tuberculosis, which may serve as an innovative basis for immunotherapy.

## Results

### MSCs phagocytose *M. tuberculosis* (Mtb)

**Figure 1:**
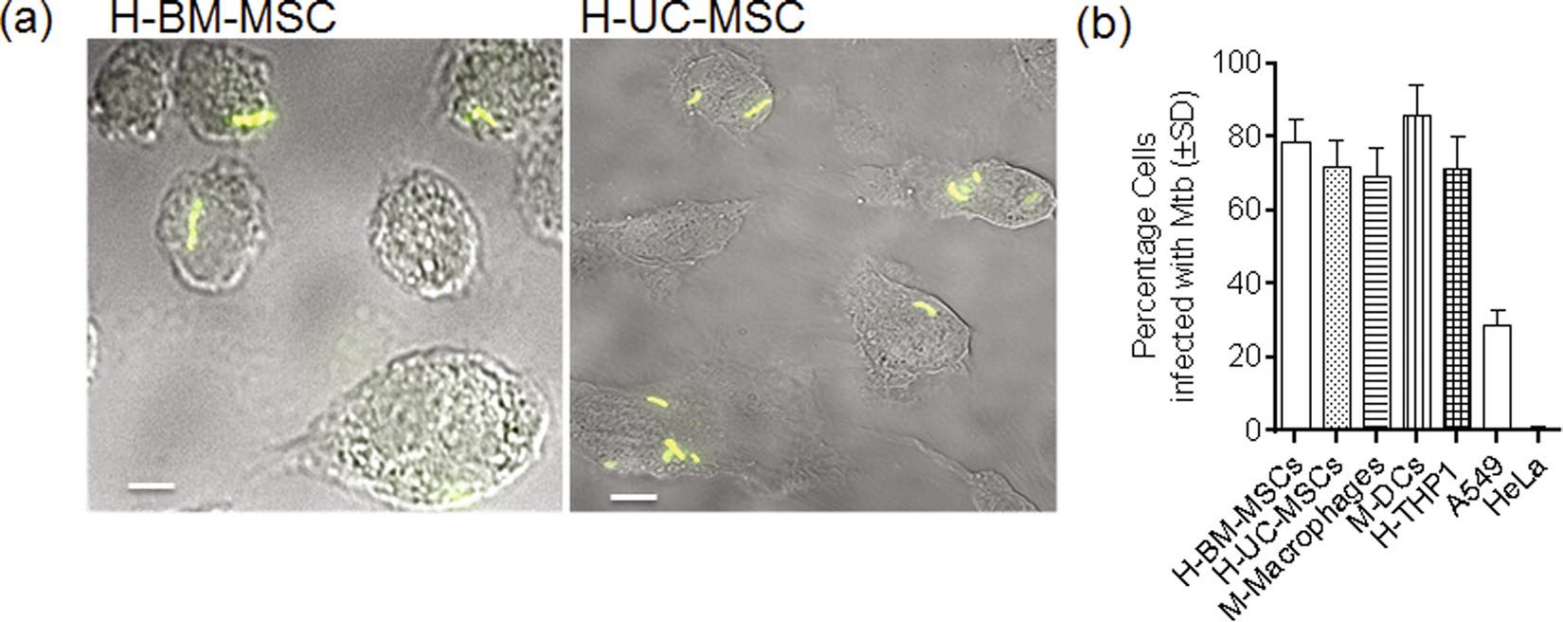
Human mesenchymal stem cells phagocytose *Mycobacterium tuberculosis (H37Rv)*. (**a**) Mesenchymal stem cells (MSCs) (fluorescent phase contrast images shown) were purified from human bone marrow (H-BM-MSC; aka BM-MSC) or umbilical cord (H-UC-MSC) and infected with virulent *gfp* labeled *Mycobacterium tuberculosis* (*gfp*Mtb) for 4 hr and washed, and the percentage of cells with intracellular bacteria analyzed using microscopy (white bar = 5 µM). (**b**) Quantification of Mtb uptake by BM-MSCs compared with mouse (M) phagocytes purified from the bone marrow of C57Bl/6 mice, human (H) THP-1 macrophages, A549 human lung epithelial cell line and HeLa cells.

Mtb is phagocytosed by macrophages and DCs through a variety of receptors such as Fc, complement receptor CR-1, C-type lectin receptors (DC-SIGN, dectin-1, Mincle and mannose receptor or MR) and scavenger receptors (SRs)^23–25^. However, MSCs have been reported to express only some types of SRs and MRs, whereas the distribution of most other receptors remains unclear^26,27^. Phagocytes, including macrophages and DCs, internalize particulate material (>1 µM) such as mycobacteria through phagocytosis, in which the plasma membrane invaginates, enclosing mycobacteria within a phagosome. In contrast, soluble material (<1 µM) is internalized through endocytosis^28^. First, to verify phagocytosis of Mtb, MSCs were purified from human bone marrow (BM-MSCs) and umbilical cord (UC-MSCs). They were incubated with *gfp*-labeled Mtb (*gfp*Mtb) at an MOI of 1. For comparison, mouse bone marrow derived macrophages, DCs, phorbol myristyl acetate-activated human THP-1 macrophages were tested. Since all these cells are highly phagocytic, the human lung epithelial cell line A549 and HeLa cells were used as additional controls. Uptake of mycobacteria was quantified through microscopy. Figure 1a,b illustrates that BM-MSCs and UC-MSCs both internalized *gfp*Mtb as efficiently as mouse and human phagocytes. Similarly, MSCs phagocytosed *gfp-*labeled *M. bovis* BCG organisms (Supplemental Fig. S1). Microscopy showed that nearly all MSCs contained Mtb 4 hr after infection, and each cell contained between 1 and 5 CFUs (Supplemental Fig. S2). In contrast, A549 was poorly phagocytic and HeLa cells did not internalize Mtb (Fig. 1b). MSCs did not show morphological changes until day 3 after Mtb infection, although some tended to fuse and form multinucleate cells 5 days after infection (Supplemental Fig. S2).

### Scavenger receptors (SRs) mediate uptake of *M. tuberculosis* in MSCs

**Figure 2:**
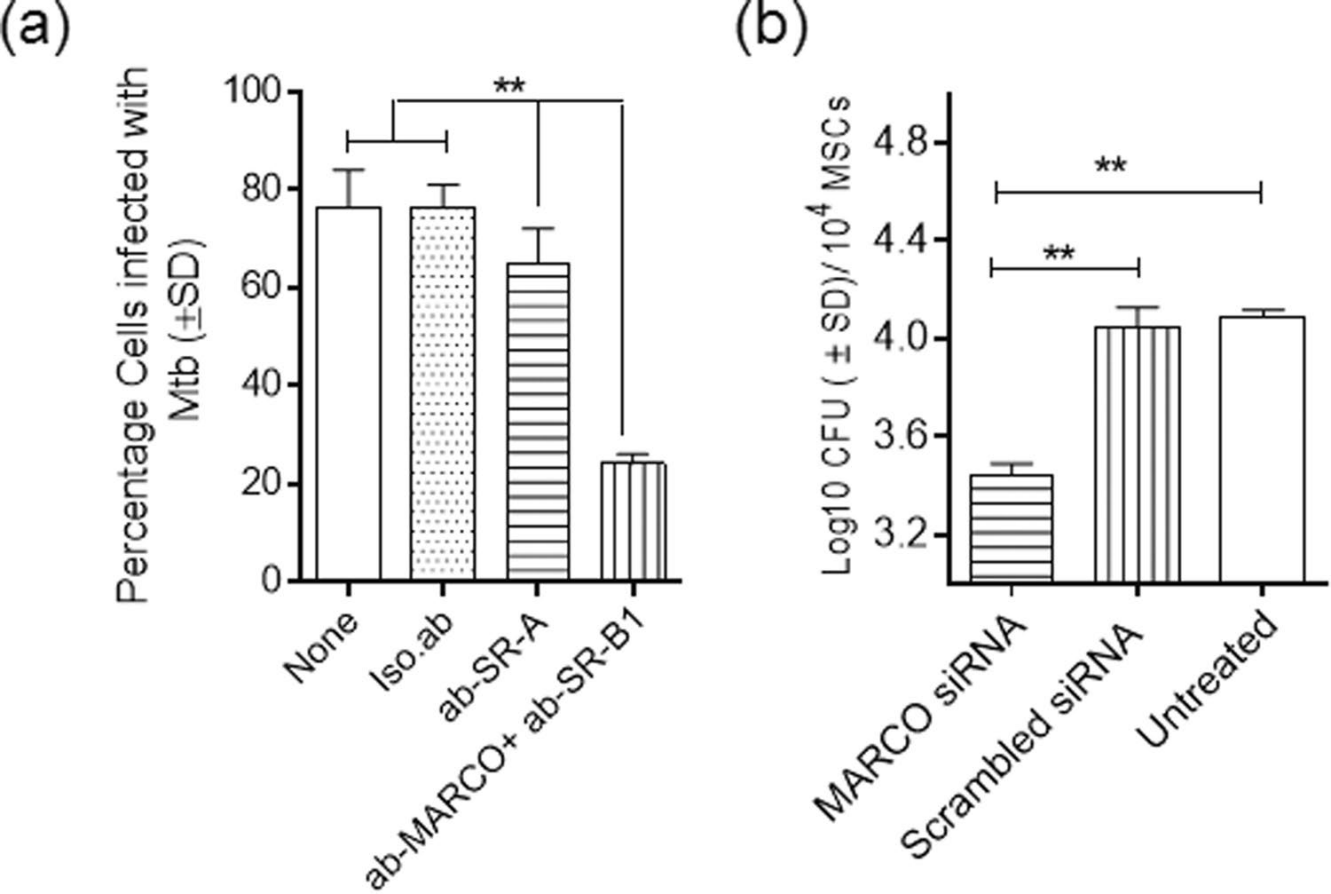
Mesenchymal stem cells phagocytose *Mycobacterium tuberculosis* through scavenger receptors. (**a**) BM-MSCs were treated with antibodies specific for scavenger receptors or isotype antibody, then infected with *gfp*Mtb. Phagocytosed bacteria were quantitated 4 hr later using microscopy. A combination of antibodies to ‘macrophage receptor with collagenous structure (MARCO) and SR-B1 (aka.CD36) inhibited uptake, whereas isotype or antibody to SR-A were not effective (**p < 0.009, ANOVA). (**b**) BM-MSCs were treated with siRNA vs. MARCO or scrambled siRNA followed by Mtb infection, and colony forming unit (CFU) counts of intracellular Mtb were determined after 4 hr uptake and after plating MSC lysates on 7H11 agar. siRNA knock down of MARCO significantly decreased uptake (**p < 0.001, ANOVA). Average CFU counts per 10^4^ MSCs of triplicate wells per group of 3 identical experiments are shown.

Among the multiple phagocyte receptors that mediate uptake of Mtb, the SRs appear to play a major role, particularly among human macrophages^25,29^. Because MSCs tend to differentiate into adipocytes and express some types of SRs, we initially hypothesized that SRs might provide a pathway for the uptake of Mtb. Earlier studies have shown that among the many types of SRs, MARCO (*macrophage receptor with collagenous structure*), SR-A (aka MSR1) and SR-B1 (aka CD36) are variably involved during the uptake of Mtb in either mouse or human macrophages^25^. AIM, a type of soluble SR, was transfected into human THP-1 macrophages and shown to decrease Mtb viability, but its role in the uptake of Mtb remains unclear^30^. Thus, we sought to characterize the roles of MARCO, SR-A and SR-B1 as potential receptors for uptake of Mtb into MSCs. All three SRs bind lipoteichoic acids from gram-positive cell walls and lipoglycans from mycobacteria^25^. When specific antibodies were used to block uptake in MSCs, antibodies against SR-A were found to be unable to inhibit uptake of Mtb (Fig. 2a). However, MSCs treated with a combination of antibodies against MARCO and SR-B1 markedly inhibited the uptake of Mtb. This result is consistent with a similar previous observation of the blockade of SRs and the uptake of Mtb in human macrophages^29^.

Previous studies have shown that CD36 binds to mycobacterial mannose-capped lipoarabinomannan (manLAM)^25^. However, macrophages from CD36^−/−^ mice are able to control the intracellular growth of both Mtb and *M. marinum* ^31^. In contrast, a polymorphism in human MARCO has been found to be associated with susceptibility to tuberculosis in the Gambian and Han Chinese populations^32,33^. Thus, we sought to confirm the role of MARCO by using siRNA knockdown in MSCs and determining CFU counts for intracellular Mtb. The results in Fig. 2b confirmed that siRNA knockdown of MARCO led to a significant decrease in the uptake of Mtb, whereas scrambled siRNA had no effect.

### MSCs endocytose oxidized-low density lipids (oxLDL) through scavenger receptors

**Figure 3:**
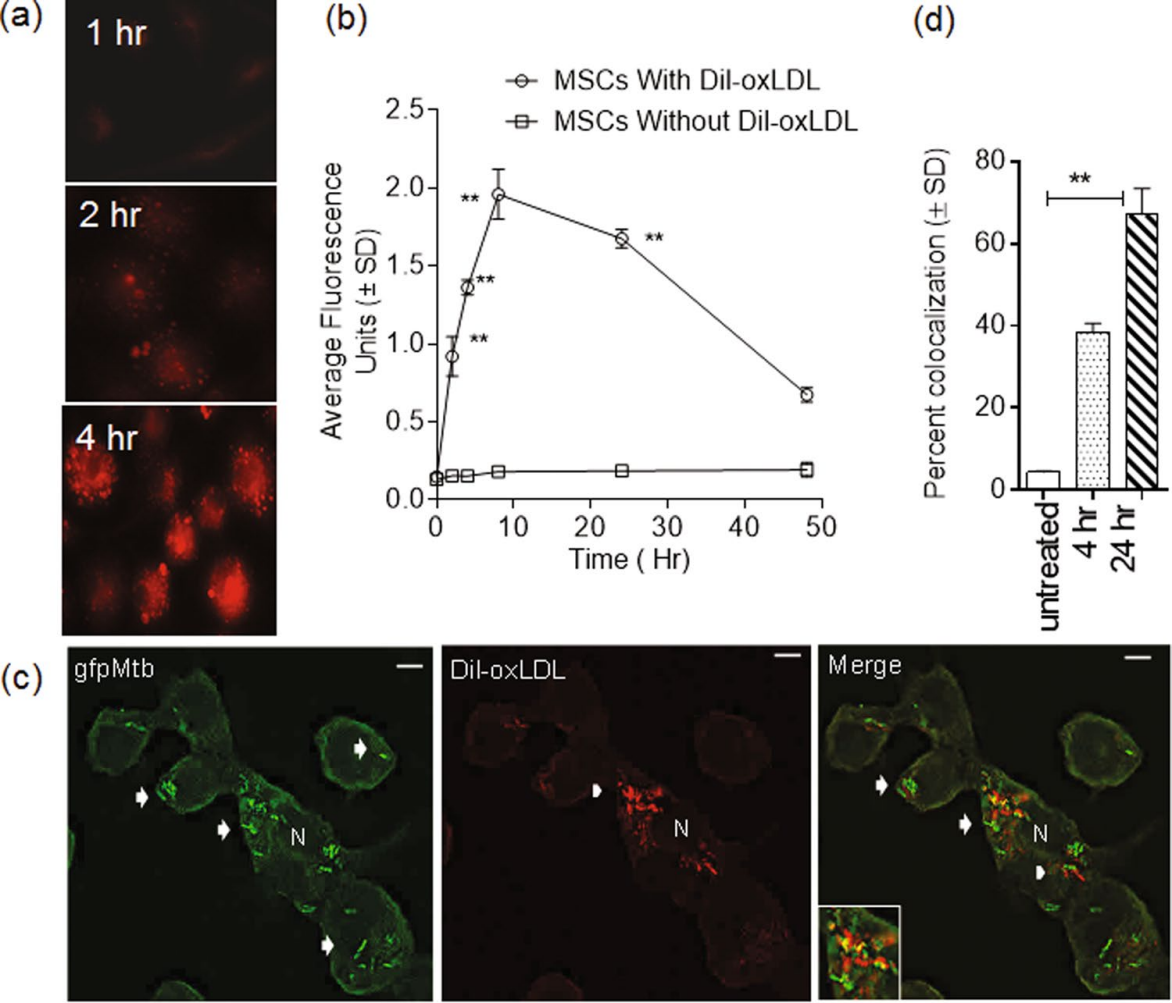
Mesenchymal stem cell endocytose low density lipoprotein (ox-LDL) through scavenger receptors, and internalized ox-LDL colocalizes with phagosomes of *gfp*Mtb. (**a**,**b**) Naïve MSCs were incubated with red fluorescent Dil-labeled oxidized low density lipoprotein (LDL) ligand (Dil-oxLDL) for SRs. At the time points indicated, the cells were chased with warm medium, and qualitative and quantitative fluorescence readouts were determined. (**a**) Fluorescence deconvolution images examined using Nikon microcopy (NIS-Elements AR deconvolution software) show endosomes containing Dil-oxLDL. (**b**) MSCs were read for intracellular fluorescence using an Ascent fluorometer (530nm-ex/599nm-em) and average fluorescence units (AFL) were plotted using triplicate wells per group in 3 replicate experiments. The uptake of Dil-oxLDL increased over 24 hr and then declined. (**c**) MSCs were treated with Dil-oxLDL for 90 min, washed and infected with *gfp*Mtb (MOI = 1), and incubated for 4 or 24 hrs. Cells were washed, fixed and imaged using deconvolution. The percentage of MSCs containing *gfp*Mtb phagosomes colocalizing with Dil-oxLDL (arrows; inset) was determined, and representative images shown are from one of three identical experiments, each of which counted and averaged 150 cells (N = nucleus; bar = 5 µM). (**d**) Quantification of MSCs showing Dil-oxLDL and *gfp*Mtb colocalization (**p < 0.009, *t* test).

SRs are traditionally involved in the uptake of lipids and lipoproteins, performing a scavenging role^34^. SRs also bind bacterial ligands and have been shown to mediate phagocytosis^29,35,36^. MARCO, SR-A and SR-B1 (CD36) can internalize oxLDL in macrophages^37,38^. To confirm that SRs were functional in MSCs, we examined endocytosis of lipids by using a fluorescent ligand, Dil-labeled oxidized low-density lipoprotein (Dil-oxLDL), a known endocytic marker for SRs in human macrophages^34^. MSCs were examined by fluorescence microscopy using a Nikon microscope with NIS-Elements deconvolution software, and fluorometry was used for quantification of uptake. Figure 3a shows that Dil-oxLDL localized to vesicles in MSCs, and Fig. 3b demonstrates a rapid uptake of Dil-oxLDL into MSCs, which declined over the course of 50 hr.

### Endocytosed Dil-oxLDL colocalizes with phagocytosed *M*. *tuberculosis* within MSCs

Many studies have shown that lipids accumulating within macrophages affect the growth, survival and persistence of intracellular Mtb^39^. Interestingly, *in vitro* loading of guinea pig macrophages with oxLDL affects the growth of Mtb^40^. Because MSCs efficiently endocytosed Dil-oxLDL, and serum is rich in LDLs, we hypothesized that the lipid-containing endosomes might fuse with the phagosomes of Mtb and in turn affect bacterial survival. Thus, MSCs were first treated with Dil-oxLDL for 90 min, and cells were then rinsed with tissue culture medium and allowed to phagocytose *gfp*Mtb. The cells were then fixed and examined under a deconvolution microscope, and the percentage of MSCs containing *gfp*Mtb phagosomes (green) colocalizing with the lipid (red) was calculated. Figure 3c illustrates that *gfp*Mtb within MSCs strongly colocalized with red fluorescent Dil-oxLDL, and Fig. 3d presents the quantification of colocalization. The time-dependent colocalization of Dil-oxLDL with *gfp*Mtb is also illustrated in Supplemental Fig. S2.

### Rapamycin up-regulates endocytosis of Dil-oxLDL through SRs by MSCs, and lipids colocalize with autophagosomes

**Figure 4:**
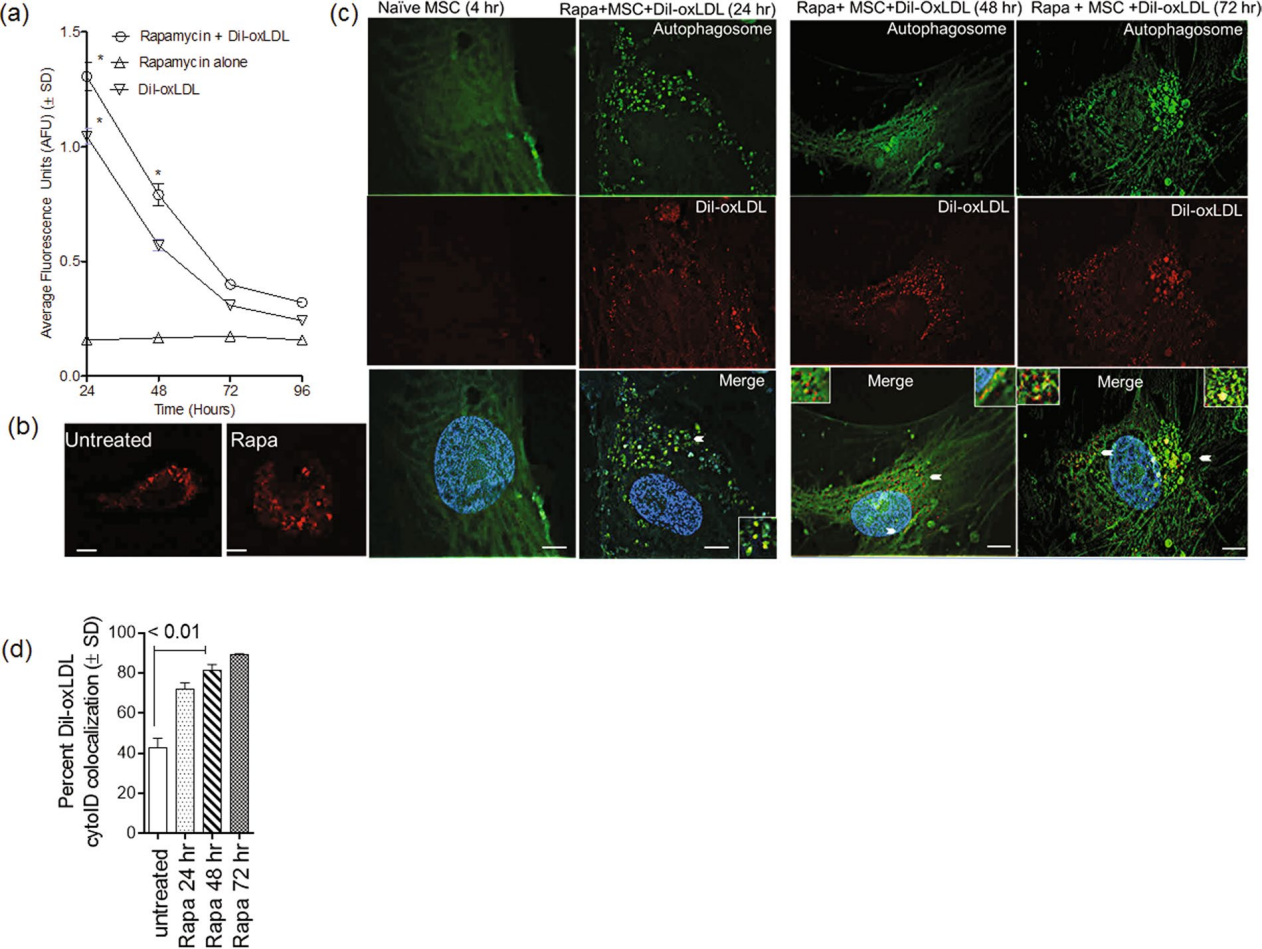
Rapamycin enhances endocytosis of Dil-oxLDL in mesenchymal stem cells and targets them to autophagosomes. (**a**) BM**-**MSCs plated in 96 wells were either tested naive or activated with 1 µM of rapamycin for 3 hr before treatment with Dil-oxLDL (100 µg/mL). Cells were washed with warm medium at the indicated times and imaged or read for fluorescence uptake at 530/590 nm (Ascent fluorometer). AFUs were plotted against time (*p < 0.01 vs. rapamycin alone; 2 similar experiments, *t* test). (**b**) Deconvolution images of Dil-oxLDL (red) within MSCs shown before and after rapamycin activation. (**c**) MSCs were activated with rapamycin (1 µM) and added with Dil-oxLDL after 90 min, washed and incubated with freshly added rapamycin. At 4, 24, 48 and 72 hr, the cells were stained using cyto-ID for autophagosomes and imaged using deconvolution microscopy. Representative images from 50 cells (triplicates) analyzed from three similar experiments are shown. Naïve MSCs containing Dil-oxLDL and autophagic puncta are shown in Supplemental Fig. S4. Rapamycin induced Dil-oxLDL (red) to colocalize with autophagosomes (inset). Nuclei are stained blue using DAPI (bar = 5 µM). (**d**) Quantitation of colocalization of Dil-oxLDL with autophagic puncta is shown.

At least three types of SRs mediate uptake of Mtb in macrophages, and we and others have previously demonstrated that expression of MARCO and SR-A (aka MSR1) is regulated by autophagy^36,41–43^. Because MSCs express SRs, we hypothesized that functionally intact SRs can endocytose Dil-oxLDL and target the lipid to autophagosomes, which then fuse with lysosomes. Moreover, we hypothesized that, if Mtb phagosomes colocalized (fused) with Dil-oxLDL-containing endosomes (as shown in Fig. 3c,d), autophagy would deliver Mtb and the lipid to lysosomes. Thus, MSCs were either tested naïve or activated with 1 µM of rapamycin for 3 hr before addition of Dil-oxLDL (100 µg/mL) and incubated. Cells were washed with warm medium, read for fluorescence uptake in a fluorometer, and analyzed using deconvolution microscopy for red colored Dil-oxLDL and cyto-ID, a green cationic amphiphilic tracer dye that labels autophagosomes. Both cyto-ID and an antibody against LC3, a marker for autophagosomes, labeled similar puncta in rapamycin-activated MSCs (Supplemental Fig. S3). Figure 4a,b illustrates that both untreated and rapamycin-activated MSCs internalized red fluorescent Dil-oxLDL, and prior activation with rapamycin enhanced the lipid uptake (Fig. 4d). In addition, fluorescence images confirmed that endosomes containing Dil-oxLDL colocalized with autophagosomes in both untreated (Supplemental Fig. S4) and rapamycin-treated MSCs (Fig. 4c).

The above studies suggested that MSCs express functional SRs that are involved in the uptake of Mtb (Fig. 2) as well as Dil-oxLDL (Figs 3 and 4). Once endocytosed, Dil-oxLDL localizes to endosomes that are able to fuse with phagosomes of Mtb, thus providing a potential source of lipids for their growth. Because oxLDL is a trigger for autophagy in macrophages, it appears possible that lipid-laden Mtb phagosomes could be delivered to lysosomes through autophagy in MSCs^44,45^.

### MSCs mediate endocytosis through the mannose receptor (MR; CD206), but MR is not involved in the uptake of *M*. *tuberculosis*

**Figure 5:**
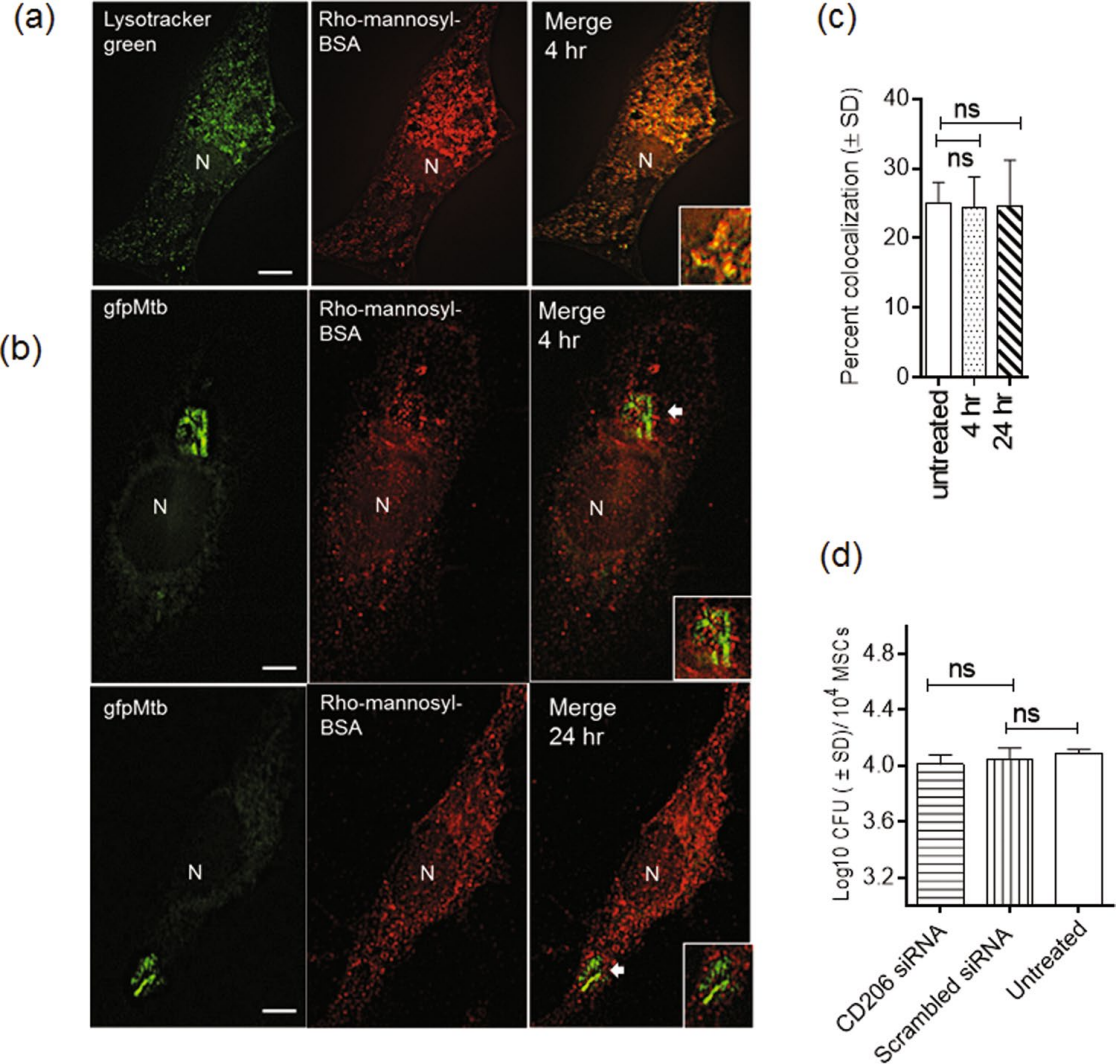
Mesenchymal stem cells mediate endocytosis through Mannose receptor (MR), but MR is not involved in the uptake of *M. tuberculosis*. (**a**) BM-MSCs were treated with LysoTracker green for 60 min, washed and incubated with rhodamine labeled mannosyl-BSA (rMBSA), a ligand for MR for 60 min. MSCs were washed and imaged using deconvolution. rMBSA colocalized strongly with LysoTracker, thus suggesting lysosomal localization for rMBSA. (**b**,**c**) BM-MSCs were incubated with rMBSA for 60 min, washed and infected with *gfp*Mtb for 4 hr. Washed MSCs were imaged at 4 and 24 hr post infection using deconvolution, and the percentage MSCs was quantified for colocalization. Images show limited colocalization (inset) of *gfp*Mtb with rMBSA and bar graph shows quantification. (**d**) BM-MSCs were treated with siRNA vs. MR or scrambled siRNA control, then infected with Mtb and CFU counts of lysates on 7H11 agar. siRNA knockdown of MR does not affect uptake of Mtb. Blot shows that siRNA knockdown decreased cytosolic MR levels. One of three similar experiments is shown.

MSCs express MR (CD206) and endocytose FITC-dextran, whereas we have demonstrated that human THP-1 macrophages endocytose fluorescently labeled mannosyl-BSA through MR^46,47^. Interestingly, MR is also a major receptor for phagocytosis of Mtb in macrophages and DCs^25,48^. To examine MR-mediated endocytosis, we incubated MSCs with the lysosome-specific tracer LysoTracker green for 60 min, then washed cells with medium and treated them with rhodamine-labeled mannosylated BSA (rMBSA). After a 60 min incubation, MSCs were examined using deconvolution microscopy. LysoTracker green strongly colocalized with rMBSA, thus confirming its lysosomal localization (Fig. 5a). Quantification was not necessary, because there was a strong colocalization of ligands. To determine whether MR was involved in phagocytosis of Mtb, MSCs were first incubated with the MR specific ligand rMBSA for 60 min, and cells were then washed and incubated with *gfp*Mtb. After 4 and 24 hr, MSCs were washed and fixed, and numbers of *gfp*Mtb phagosomes colocalizing with rMBSA were analyzed using deconvolution microscopy. Figure 5b,c shows that MSCs internalized the ligand of MR, but *gfp*Mtb did not substantially colocalize with rMBSA.

These observations suggested that MSCs show active endocytosis of mannosylated ligands but may not phagocytose Mtb through MR. MSCs were then treated with siRNA against MR (CD206), then infected with Mtb and CFU counts of MSC lysates were performed at 4 hr. Figure 5d demonstrates that siRNA knockdown of MR had no significant effect on Mtb uptake.

### Intrinsic autophagy in MSCs decreases the viability of intracellular *M. tuberculosis*

**Figure 6:**
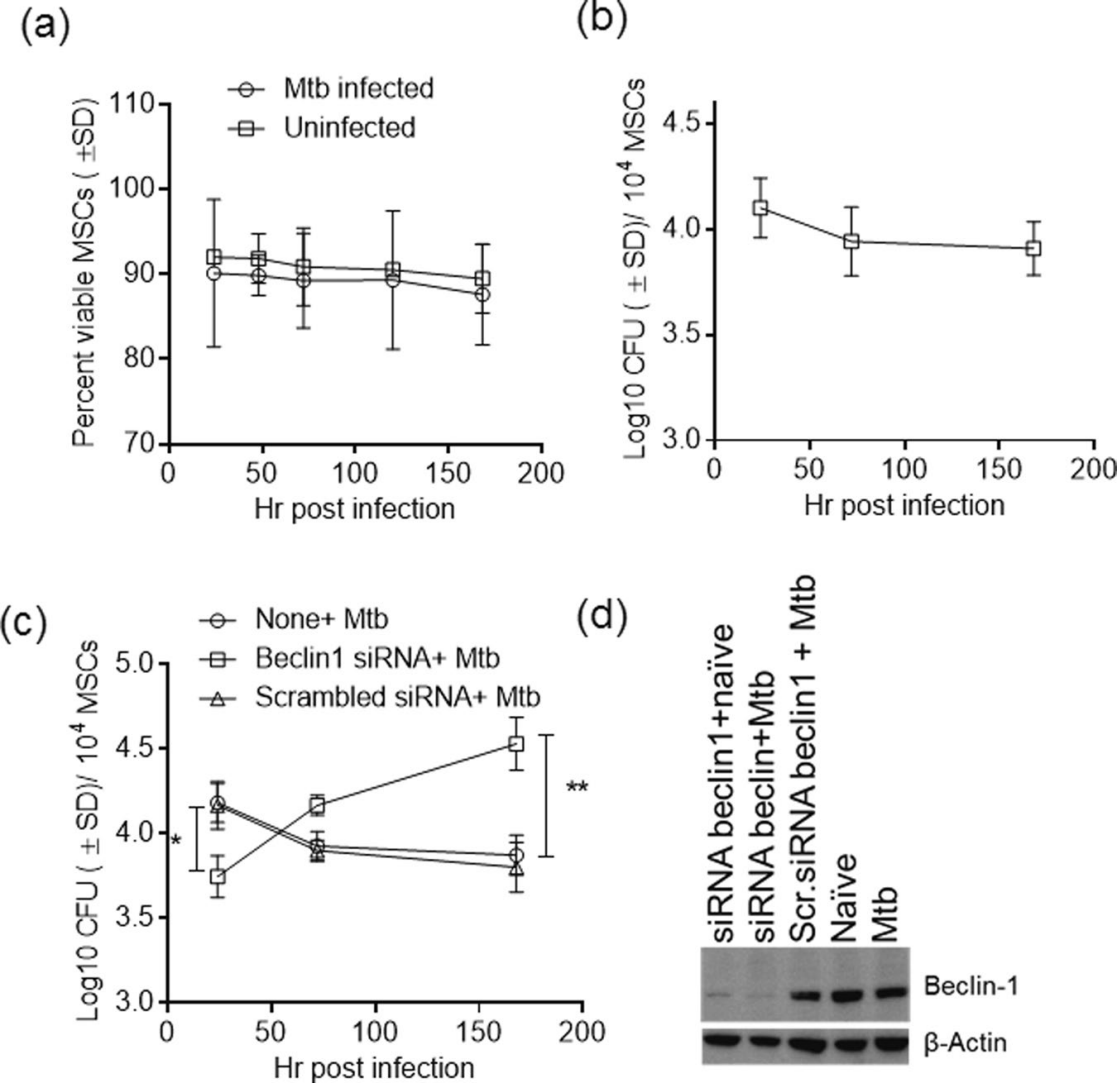
siRNA vs.beclin-1 mediated inhibition of autophagy in mesenchymal stem cells enhances survival of intracellular *M. tuberculosis*. Untreated or treated BM-MSCs were infected with Mtb (MOI = 1). Lysates were plated for viability of intracellular Mtb by plating lysates on 7H11 agar plates at the indicated time intervals, and stem cell viability was evaluated using alamar blue. (**a**) Mtb infected MSCs retained approximately 90% viability over 7 days of culture. (**b**) Mtb counts of MSCs did not increase over the course of 7 days of culture. (**c**) BM-MSCs were treated with siRNA vs. belcin-1 or scrambled siRNA control, then infected with Mtb and CFU counts done  over the course 7 days. Average CFU counts per 10^4^ MSCs of triplicate wells per group of 3 identical experiments are shown in panels b and c (**p < 0.005,*p < 0.05, ANOVA). siRNA vs.beclin-1 decreased the initial uptake of Mtb, although intracellular Mtb increased in numbers after knockdown of beclin-1. (**d**) MSC lysates were tested for beclin-1 protein levels using western blotting to confirm siRNA knockdown.

When MSCs were infected with Mtb, they remained healthy for more than 7 days during *in vitro* culture and showed no signs of either apoptosis or loss of viability (Fig. 6a). Furthermore, Mtb did not multiply within MSCs (Fig. 6b,c) but instead showed a decline in numbers over the course of 7 days. In contrast, parallel cultures of PMA-activated THP-1 macrophages supported rapid growth of Mtb over the course of 7 days (Supplemental Fig. S5). In addition to nitric oxide and reactive oxygen radical-mediated killing, phagosome-lysosome (PL) fusion also causes loss of viability of Mtb in macrophages^7^. Mycobacterial phagosomes are usually delivered to lysosomes in macrophages through a *rab*- and SNARE-protein-dependent PL fusion pathway^4,49^. However, recent studies have indicated that *atg-*gene-dependent autophagy is an emerging intrinsic mechanism of macrophages that enables fusion of mycobacterial phagosomes to lysosomes^50^. Indeed, we and others have demonstrated that autophagy kills intracellular Mtb (Supplemental Fig. S5) and *M. bovis* BCG^42,51,52^. Interestingly, intrinsic autophagy has been found to occur in mesenchymal stem cells and is thought to be essential for their self-renewal, pluripotency, differentiation and quiescence^53^. We therefore examined the initial hypothesis that low-level intrinsic autophagy in MSCs restricts the growth of Mtb. Because beclin-1 is an initiator of autophagy, siRNA against beclin-1 was used to blockade autophagy^42,51,52^. The growth of Mtb was significantly enhanced after siRNA knockdown of beclin-1 (Fig. 6c).

Interestingly, siRNA against beclin-1, but not scrambled siRNA, decreased the initial uptake of Mtb during phagocytosis (Fig. 6c). However, western blot analysis confirmed that the levels of beclin-1 protein were decreased after siRNA knockdown but not after treatment with scrambled siRNA (Fig. 6d). Because beclin-1 is recruited into the phagocytic cup during phagocytosis, beclin-1 may be a regulator of phagocytosis that also affects uptake of Mtb^54^. However, beclin-1 knockdown did not decrease but instead increased the subsequent growth of Mtb over the course of 7 days (Fig. 6c) This result is consistent with the notion that blockade of receptor-mediated entry of Mtb in human macrophages has no significant effect on its subsequent multiplication^29^.

### Rapamycin-induced autophagy in MSCs decreases the viability of intracellular *M. tuberculosis*

**Figure 7:**
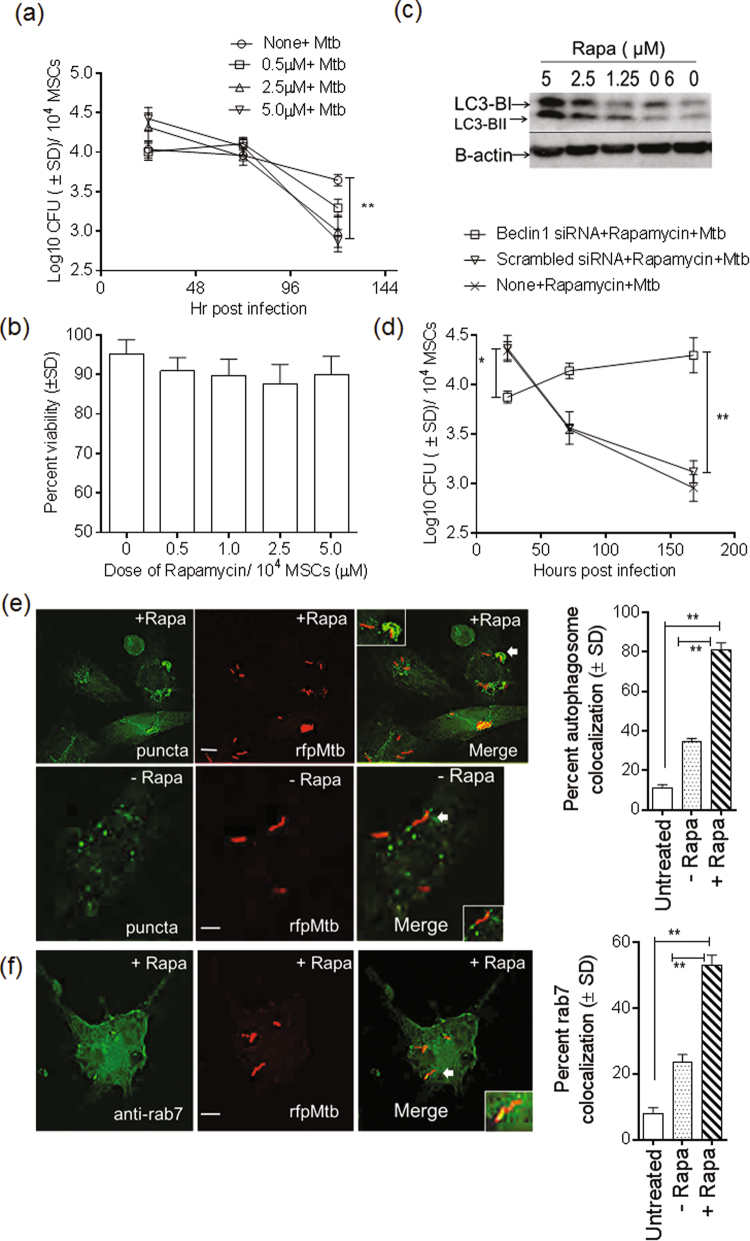
Rapamycin enhances autophagy mediated killing of intracellular *M. tuberculosis* in mesenchymal stem cells. Untreated or rapamycin (µM doses indicated) treated BM-MSCs were infected with Mtb (MOI = 1). Lysates were plated on 7H11 agar for viability of intracellular Mtb, and stem cell viability evaluated using alamar blue. (**a**) Rapamycin caused a dose-dependent loss of viability of Mtb within MSCs. Data from one of three similar experiments are shown (**p < 0.007, 5 µM dose vs. none; ANOVA). (**b**) Rapamycin treated BM-MSCs retained approximately 90% viability on day 7. (**c**) MSC lysates on day 1 post rapamycin treatment were tested for microtubule associated with light chain (LC3) lipidation by using western blotting. (**d**) To confirm the specificity of rapamycin effects, MSCs were treated first with siRNA vs. beclin-1 or scrambled control followed by rapamycin (5 µM dose), and CFU counts of Mtb were determined by plating MSCs lysates. (**e**) MSCs were treated with rapamycin (5 µM), and after a 4 hr infection with *rfp*Mtb, MSCs were fixed on day 1 and stained for autophagosomes using cyto-ID or an antibody to rab7 lysosomal marker. Images were analyzed using deconvolution microscopy and quantified. Images show that rapamycin induces strong colocalization of autophagosomes with *rfp*Mtb (inset) compared with untreated cells (white bar = 5 µM). Bar graph shows data from three experiments of the percentage MSCs showing colocalization of *rfp*Mtb with autophagic puncta. (**f**) Rapamycin also induces stronger staining of *rab*7 on *rfp*Mtb phagosomes (inset) thus suggesting lysosomal fusion. Bar graph indicates quantification.

Because intrinsic autophagy restricted the growth of Mtb, we hypothesized that enhancing autophagy would be more effective in killing intracellular Mtb, thus perhaps paving the way to immunotherapy for tuberculosis. MSCs were therefore activated with varying doses of rapamycin, and the viability of Mtb was evaluated. Rapamycin had a dose-dependent effect and decreased the numbers of Mtb, the effective range being 1–5 µM (Fig. 7a). Such MSCs remained approximately 90% viable (Fig. 7b). Rapamycin also enhanced killing of intracellular *M. bovis* BCG in a range of 2.5–5 µM (Supplemental Fig. S7 *)*. Activation of autophagy in macrophages with rapamycin induces the lipidation of microtubule-associated light chain-3 (LC3)^52^. To confirm the effects of rapamycin, day 1 MSC lysates obtained after drug treatment (Fig. 7a) were analyzed using a western blot and antibodies against LC3. Rapamycin induced a stronger lipidation of LC3 (Fig. 7c). In additional experiments, MSCs were treated with siRNA against beclin-1, then subjected to rapamycin activation and Mtb infection, and CFU counts of MSC cell lysates were determined over 7 days. Figure 7d confirms that rapamycin-induced killing of Mtb in MSCs was inhibited by siRNA knockdown of beclin-1. Finally, rapamycin-induced autophagy was validated using cellular trafficking of Mtb phagosomes^52^. Unactivated or rapamycin-activated MSCs were infected with *rfp*Mtb and were analyzed using deconvolution microscopy after staining with cyto-ID, which labels autophagosomes, or by fixing cells and staining using an antibody to *rab7* that labels lysosomes and autophagolysosomes^52^. Imaging (color panels) and quantification of colocalization (bar graph) (Fig. 7e) illustrated that fewer autophagosomes colocalized with *rfp*Mtb in untreated MSCs, but their numbers increased after rapamycin treatment. Likewise, an antibody to *rab7* showed more labeling of *rfp*Mtb phagosomes among rapamycin treated MSCs (Fig. 7f). It is well established that during autophagy, the p62 and NBR1 substrates are depleted in autophagolysosomes^55^. In additional control experiments, MSCs were treated or not treated with rapamycin and siRNA against beclin-1, and cells were then lysed. Sandwich ELISA of MSC lysates confirmed that p62 and NBR1 levels declined after induction of autophagy with rapamycin (Supplemental Fig. S8 *)*.

### MSCs secrete nitric oxide after *M. tuberculosis* infection

**Figure 8:**
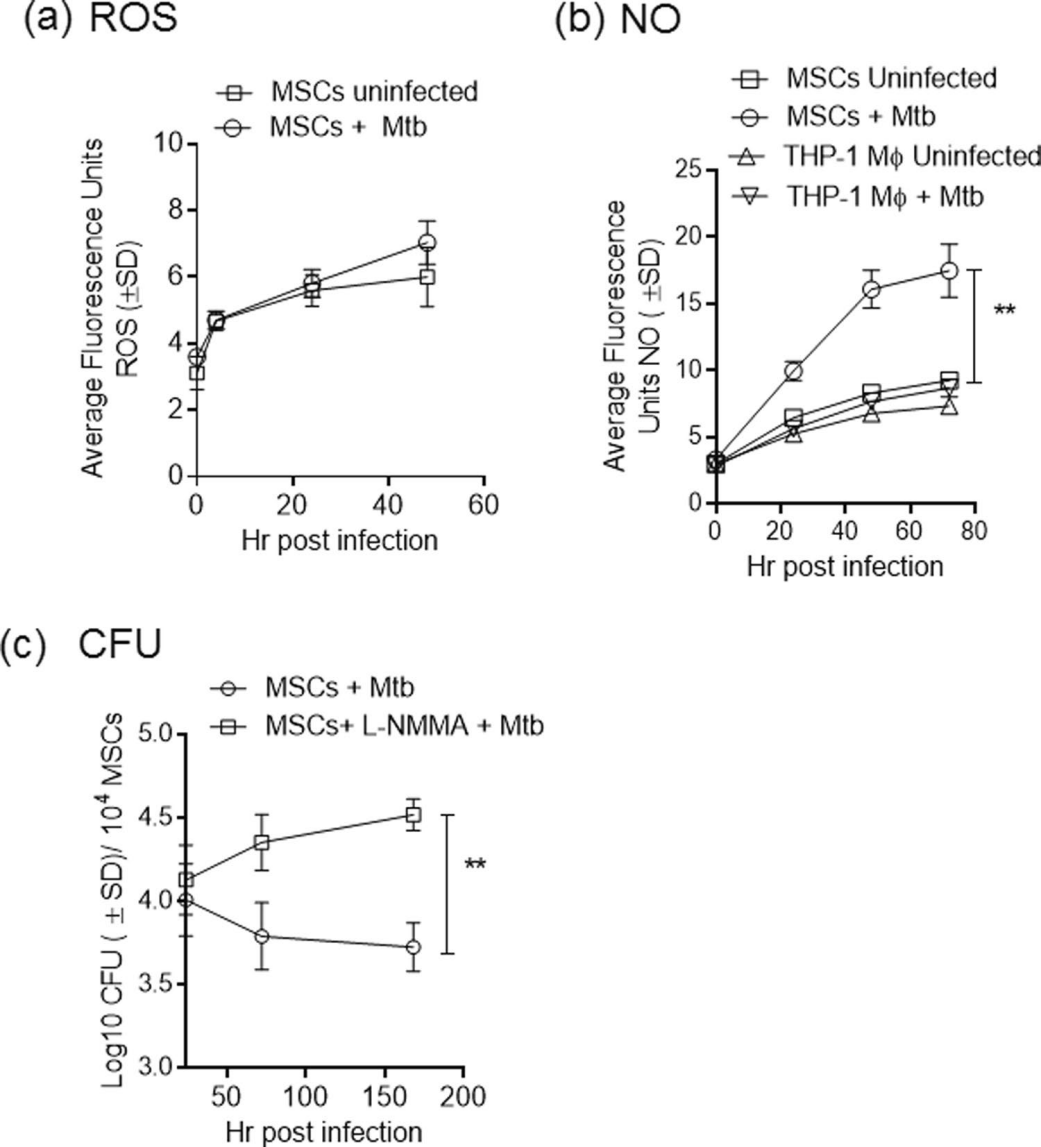
Mesenchymal stem cells release nitric oxide after infection with *M. tuberculosis* and inhibition of NO increases viability of intracellular organisms. BM-MSCs or human THP-1 macrophages were infected with Mtb (MOI = 1) and tested for the release of oxidants by using DCFDA (5-(and-6)-Carboxy-2′, 7′-Dichlorofluorescein Diacetate) for the detection of reactive oxygen species (ROS) and DAF2-DA (Diaminoflurescein diacetate) for nitric oxide with a fluorometer in three similar experiments. (**a**) MSCs secreted limited levels of ROS with or without infection. (**b**) Mtb infected MSCs secreted elevated levels of NO compared with similarly infected THP-1 macrophages (**p < 0.05, *t* test). (**c**) MSCs were incubated in 100 µM N (G)-monomethyl-L-arginine (L-NMMA) after infection with Mtb, and cell lysates were plated for CFUs over time. Blockade of NO synthesis enhanced intracellular survival of Mtb (**p < 0.009, *t* test).

In addition to PL fusion, which leads to Mtb being killed in lysosomes, oxidants are released by phagocytes in response to mycobacterial infection^56,57^. Because oxidants can kill mycobacteria, MSCs were analyzed for reactive oxygen species (ROS) and nitric oxide (NO) levels after Mtb infection, by using fluorescent probes. MSCs secreted low levels of ROS after infection (Fig. 8a) However, compared with similarly infected THP-1 macrophages, MSCs released higher levels of NO, as detected by the fluorescent DAF2-DA probe (Fig. 8b)^57^. Because human THP-1 and PBMC-derived macrophages release low levels of NO, it has been proposed that the anti-mycobacterial effects of NO in humans may be marginal^58^. To validate the role of NO, MSCs were infected with Mtb and incubated in the presence of the NO inhibitor N (G)-monomethyl-L-arginine (L-NMMA). Figure 8c demonstrates that inhibition of NO levels by L-NMMA enhanced the intracellular viability of Mtb.

## Discussion

This study supports the emerging concept that MSCs are novel phagocytic cells that, like macrophages, can internalize Mtb. Since MSCs were present in Mtb containing granulomas of mice and humans, these cells have been thought to play a role during the pathogenesis of tuberculosis^19,20^. Macrophages are a key component of granulomas, which wall off the lesions of tuberculosis^10^. Here, we present the first evidence that MSCs are phagocytic and demonstrate an innate control of mycobacterial replication through intrinsic autophagy and secretion of oxidants.

Although MSCs have been found to contain Mtb, the mechanisms of Mtb uptake have remained unclear. Unlike macrophages, which express multiple surface receptors, MSCs are derived from the mesoderm and do not differentiate into hematopoietic cells. However, they are multipotent, with an ability differentiate into adipocytes, neuronal cells and osteoclasts^59^. Fewer receptors are therefore known on MSCs, although, because of the propensity of these cells to differentiate into adipocytes, they have been found to express SRs and endocytose lipids^60^. At least one study has shown that they can endocytose FITC-dextran through MR^46^.

SRs are a unique class of lipid-endocytosing receptors that are also known to bind mycobacterial lipids and glycoproteins^25^. As a consequence, at least three SRs (MARCO, SR-A and SR-B1) are known to mediate uptake of Mtb. Interestingly, mouse and human macrophages behave differently after SR-mediated uptake of Mtb. Thus, human MARCO is associated with resistance to Mtb, whereas CD36^−/−^ (SR-B1) mouse macrophages do not show a phenotype in regulating mycobacterial growth^31,32^. In this study, consistently with previous observations on SRs of macrophages, we found that both MARCO and SR-B1 were involved during the uptake of Mtb by MSCs, because antibody-mediated blockade decreased mycobacterial uptake, and siRNA knockdown experiments confirmed the role for MARCO (Figs 1 and 2). Paradoxically, the MR of MSCs was able to endocytose rMBSA but was not involved in the uptake of Mtb (Fig. 5). These results suggest that MSCs use at least two types of SRs to internalize Mtb, although the role of other receptors cannot be ruled out.

Intriguingly, after phagocytosis, Mtb did not replicate within MSCs. Because the MSCs were not activated, an intrinsic mechanism of regulating mycobacterial growth was apparent (Fig. 6). In macrophages, phagocytosed mycobacteria can be sorted to lysosomes, and phagosome-lysosome fusion kills Mtb. In addition, oxidants (ROS, NO) released in response to infection can also kill Mtb^61^. Autophagy is another emerging innate mechanism of phagocytes that has been found to affect the viability of multiple pathogens, including Mtb, through fusion of autophagosomes with lysosomes. Autophagy is regulated by multiple *atg* genes and is induced by stress factors including mycobacterial infection^4,62^. We and others have previously demonstrated that autophagy decreases mycobacterial viability in macrophages^50,52^. Interestingly, autophagy appears to be an intrinsic property of many types of stem cells and is thought to be essential for their self-renewal, pluripotency and differentiation^53^. Our studies showed that the growth of Mtb is restricted by intrinsic autophagy even in unactivated MSCs, since beclin-1 knockdown of autophagy increased Mtb survival (Fig. 6).

The involvement of SRs during phagocytosis of Mtb by MSCs suggests an intriguing role for these receptors through lipid and mycobacterial uptake. It has been reported that the Mtb-derived trehalose dimycolate lipid uses the MARCO, CD14 and TLR-2 co-receptors to enter macrophages and triggers a pro-inflammatory cytokine response, thus aiding in bacterial containment. Many studies have emphasized the importance of lipids affecting the growth of Mtb in macrophages^63,64^. Thus, host lipids have been reported to be integrated into mycobacterial triacylglycerol, which is associated with dormancy in Mtb^39,65,66^. Other studies have shown that foamy macrophages containing lipid bodies facilitate the persistence of Mtb^10,47,67^. Therefore, it seems reasonable to propose that SRs mediate lipid accumulation in macrophages and affect the growth of intracellular Mtb. Because LDLs are present in serum, the levels of expression of SRs and the local environment may ultimately determine lipid uptake and growth of Mtb. Mutations in the gene for MARCO have been reported to be associated with susceptibility to human tuberculosis^32,33^.

Intriguingly, SRs appear to have different effects on Mtb growth in macrophages than in MSCs. We have previously demonstrated that the growth of *M. bovis* BCG is increased in the organs of mice with a conditional autophagy (atg7flox mice) deficiency in myeloid cells (aka atg7^−/−^ mice)^42^. Interestingly, SRs (MARCO and SR-A; MSR1) are up-regulated in atg7^−/−^ macrophages, correlating with increased intracellular growth of BCG^42^. A recent study, however, has shown that many autophagy-gene-deficient mice are resistant aerosol-induced tuberculosis, whereas atg5-deficient mice show a neutrophil-dependent increased susceptibility to tuberculosis^68^. Nonetheless, the increased growth of *M. bovis* BCG in atg7^−/−^ macrophages is consistent with the observation that oxLDL accumulation in guinea pig macrophages increases the growth of Mtb^40^. Because oxLDL triggers autophagy in mouse macrophages^69,70^, we propose that a lack of autophagy, as observed in atg7^−/−^ macrophages, may prevent degradation of oxLDL, which may then be used by Mtb as a nutrient for growth. Unlike macrophages, however, MSCs have an intrinsic autophagy process that delivers Dil-oxLDL (Figs 3 and 4) and Mtb (Fig. 6) to autophagolysosomes. Furthermore, MSCs are different from macrophages in their oxidant response to Mtb infection. In general, mouse macrophages, compared with human macrophages, secrete more ROS and NO after Mtb infection^58^. Compared with human THP-1 macrophages, however, MSCs secreted more NO after Mtb infection, and blockade of NO enhanced the growth of Mtb (Fig. 8). Thus, MSCs appear to restrict the growth of Mtb through a combination of intrinsic autophagy and NO-mediated effects and are different from macrophages.

Although autophagy has been reported in MSCs as a self-regulating mechanism, we showed a novel molecular connection between intrinsic autophagy and decreased viability of Mtb^53^. This observation in turn led to the intriguing finding that rapamycin-induced autophagy in MSCs can be used to increase the killing of intracellular Mtb (Fig. 7). Unlike macrophages and many other immune cells, MSCs can be harvested from bone marrow and expanded into high numbers (>100 million per donor), as required for transplantation^71,72^. After *in vivo* seeding, they localize to multiple organs, including lungs, and self-populate. Phase I clinical studies have indicated that infusion of autologous MSCs is well tolerated by patients with MDR/XDR tuberculosis, who also show clinical improvement^22^. Because MSCs were administered along with second-line chemotherapy in these patients, improvement in lung function was probably associated with better bacterial clearance.

Because rapamycin-induced autophagy can kill even MDR-Mtb organisms (Supplemental Fig. S6), we propose that rapamycin-activated ‘conditioned’ autologous or heterologous MSCs can be used for transfusion into MDR-tuberculosis patients for the purpose of immunotherapy. A beclin-1-derived peptide that triggers autophagy has been described, and thus it may even be possible to ‘engineer’ stem cells for increased autophagy, after which they could be infused into patients for better stem-cell-mediated bactericidal treatment^73^. Alternatively, aerosolized formulations of rapamycin and anti-tuberculosis drugs are being optimized, and it appears to be feasible to infuse MSCs into MDR/XDR patients and then elicit *in situ* activation with nebulized drug combinations^74^. MSCs therefore have a potential to emerge as novel immune-therapeutic agents for tuberculosis treatment.

## Methods

### Mycobacterial strains and MSCs

The methods were carried out in accordance with the relevant guidelines and were approved under institutional biosafety protocols under IBC 12–160 and HSC-MS-15-0548. The *M. tuberculosis* (H37Rv) (ATCC-27294) strain was obtained from the ATCC and was grown in BBL Middlebrook 7H9 broth with OADC enrichment (BD Biosciences, CA, #211886) at 37 °C and 5% CO_2_. Green-fluorescent-protein-expressing *M. tuberculosis* H37Rv (*gfp*Mtb) strains and *M. bovis* BCG were constructed as previously described^52^. Red-fluorescent-protein-expressing *M. tuberculosis* H37Rv (*rfp*Mtb) was a kind gift from Dr. Malini Madiraju (University of Texas, Tyler campus, TX). All mycobacterial strains were grown for 7 days in 7H9 broth with (for *gfp, rfp* strains) or without 25 µg/mL kanamycin, and aliquots containing ~10^8^ viable bacterial cells were frozen for subsequent use. Before use, aliquots were thawed, washed three times in PBS (x12,000 rpm; 15 min), and sonicated at 4 watts with a sonicator (60 Sonic Dismembrator, Fisher Scientific) to prepare a uniform single-cell suspension of the bacteria.

### Mesenchymal stem cells

All stem cell methods and procedures described below were carried out in accordance with the procedures approved under protocols IBC 12–160 and HSC-MS-15-0548. Human bone marrow (BM)-derived and umbilical cord (UC)-derived MSCs were isolated, cultivated and characterized according to previously described protocols, using density centrifugation and plastic adherence^51^. After expansion, adherent MSCs were washed and harvested with 0.25% trypsin/1 mM EDTA treatment (Life Technologies) for 5 min at 37 °C and resuspended with fresh culture medium (α-MEM + 16% fetal bovine serum, + 10 µg/mL penicillin + 5 µg/mL gentamicin) and drug-free medium for subsequent experiments in 6/24/96-well plates or 8-chamber slides. The human macrophage cell line THP-1 was purchased from the ATCC (TIB#3456), MD, USA, and was grown and maintained at 37 °C and 5% CO_2_ in RPMI-1640 (Sigma Aldrich, St Louis, MO, USA) medium with 16 mM HEPES, 10% heat-inactivated FBS, 5 μg/mL gentamicin and 10 µg/mL penicillin. Mouse bone marrow-derived macrophages and DCs were isolated and cultured as previously described^31^. All cells were maintained in drug-free medium for various assays described herein.

### Dil-OxLDL ligand uptake and mycobacterial uptake by scavenger receptors

#### Dil-OxLDL uptake

To determine the expression of functional SRs on MSCs, fluorescent Dil-labeled oxidized LDL (Dil-oxLDL; Alpha Aesar; BT920) was added to MSCs at 100 µg/mL in 96-well plates or 8-chamber slides. Time-dependent uptake of Dil-oxLDL was measured by both fluorometry and fluorescence microscopy. Fluorometer (Fluoroskan Ascent CF, Microplate Fluorometer, Thermo Fisher Scientific)-based detection of uptake of Dil-oxLDL by MSC was performed by reading the intracellular fluorescence signal over time for untreated MSC vs. Dil-oxLDL treated MSCs plated in 96 wells. Cells were washed twice with warm medium before being read for fluorescence at ex. 530 nm/em. 590 nm, and average fluorescence units (AFUs ± SD) were plotted vs. time. To detect uptake of Dil-oxLDL by fluorescence microscopy, at indicated time points, MSCs treated with Dil-oxLDL were washed 3X with PBS, fixed with 2.7% the paraformaldehyde for 15 min and again washed 3X with PBS before examination under a microscope (Nikon Eclipse 80i) using a TRITC filter (ex. 530–560 nm, em. 570–630 nm). Analysis and deconvolution of images were analyzed as indicated below. *Dil-oxLDL colocalization with Mtb*: MSCs were treated with Dil-oxLDL for the indicated lengths of time, washed and infected with *gfp or rfp*Mtb for 4 hr. After being washed, the MSCs were incubated for various times and fixed, and colocalization of Mtb with Dil-oxLDL was determined as follows. *Colocalization of gfp or rfpMTB with either Dil-oxLDL:* In several figures, colocalization of bacterial phagosomes were analysed using green or red colored mycobacteria with either red or green colored reagents. Colocalization was defined as a clear yellow color resulting from merge of green and red colors using the real time (RT) deconvolution software integrated into the NIS-Elements software with a Nikon Eclipse N80i microscope as described us and others^52,75^. Briefly MSCs were plated at triplicate chambers and infected or treated. One to 5 bacterial phagosomes were scored for colocalization or not in each stem cell, and 50 such MSCs were counted in triplicate for a single experiment. The experiment was done three times. Thus, the mean percentage of colocalization ( ± SD) was representative of 150 cells counted and one representative experiment of three similar experiments is shown. *Dil-oxLDL colocalization with autophagic puncta:* Unlike bacterial phagosomes, Dil-oxLDL is taken up into endosomes which then fuse with autophagic puncta (Fig. 4). Colocalization of red Dil-oxLDL with green puncta stained by cytoID was determined by counting 100 puncta in each cell with 50 cells counted in triplicate chambers ( ± SD); 3 experiments were performed and one representative experiment shown^76^. CytoID is a novel dye that selectively labels autophagosomes with minimal staining of lysosomes (Enzo Life Sciences, ENZ-51031-K200).

#### Antibody-based inhibition of surface scavenger receptors

MSCs were pretreated as indicated, with anti-macrophage scavenger receptor I (A) antibody (Abcam # ab6417), anti-MARCO antibody (Abcam # ab103311), anti-scavenging receptor SR-BI antibody (Abcam # ab52629) and mouse anti-human IgG2a isotype (Abcam # ab91361) for 4 hr and were then washed three times with warm medium before Mtb was added for a 4 hr infection (MOI 1:1). Quantification of phagocytosis was then performed microscopically by counting and averaging 25 different fields that contained at least 100 cells each in duplicate chambers per experiment; the counts were performed three times. Macrophages were then lysed immediately to quantify the number of Mtb taken up after 4 hr. Mycobacterial uptake studies were conducted using MSCs, human THP1 macrophages, mouse macrophages and DCs. Human lung epithelial cell line A549 (ATCC CRM-CCL185) and HeLa cells (ATCC;CCL2) were used as additional controls.

### Rhodamine-labeled MBSA and mycobacterial uptake via MR

#### LysoTracker green and rMBSA ligand uptake by MR

LysoTracker green (Molecular Probes Inc.) was used at 100 nM for 60 min, and cells were then treated with 10 µg/mL of rMBSA (Sigma Aldrich Co. MO) for 60 min. Autophagosome puncta colocalizing with *gfp-* or *rfp*-labeled mycobacteria were scored and evaluated as above. *Mtb uptake by MR*: MSCs were treated with siRNA against MR (sc-45360; CD206) or a scrambled siRNA (sc-37007) control, then infected with Mtb. Cells were lysed immediately, and CFU counts of lysates were determined by plating cell lysates on 7H11 agar in three separate experiments. Western blot analysis was used to confirm specific knockdown of MR using antibody (Abcam).

### siRNA knockdown of MSCs and CFU assay

The kits for human MARCO siRNA, human beclin-1 (BECN1) and scrambled siRNA were purchased from Santa Cruz Biotech (sc-75747, sc-29797 and sc-37007, respectively). Cells were treated with siRNA and the scrambled control according to the manufacturers’ instructions, and this was followed by addition of Mtb (H37Rv) for 4 hr (MOI of 1:1). Cells were then lysed, and 0.1X dilutions were plated on 7H11 agar plates for CFU counts, which were obtained after 21 days of incubation. As indicated, rapamycin or L-NMMA was added in varying doses (µm). Details of the CFU assay has been described elsewhere^31^.

### Evaluation of autophagosome puncta, lysosomes, and localization of *gfp-* or *rfp*Mtb within MSCs

MSCs were plated in 8-well slide chambers at a density of 1000 cells/chamber and loaded with SR ligands or activated *in situ* with rapamycin, and cells were then rinsed with medium and treated with either *gfp-* or *rfp*-labeled Mtb at an MOI of 1:2–5. Cyto-ID was used to stain autophagosomes*. LC3 antibody for autophagosomes:* Confirmation for the staining of cyto-ID puncta as autophagosomes was conducted by incubating rapamycin-activated MSCs with cyto-ID, fixation and staining with a specific antibody for the human LC3 (Cell Signaling # 3868) followed by secondary staining with Texas Red-conjugated anti-IgG (Jackson Immunoresearch # 111-095-003). (Supplemental Fig. 8).

### Western blot and ELISA and cell viability assay

#### Western blots

Naïve or siRNA- or rapamycin- treated MSCs were infected with Mtb and lysed at intervals. Cell lysates were subjected to western blotting using antibodies specific for human LC3-I/LC3-II and beclin-1 (Cell Signaling # 8666, # 3738). Blots were then probed with horseradish peroxidase-conjugated anti-rabbit IgG as a secondary antibody (Cell Signaling # 7074) and assessed via chemiluminescence. A protein loading control of β-actin (Cell Signaling #4967) was also included. MSCs lysates were also analyzed for intracellular levels of autophagy substrates p62 and NBR1 by using ELISA kits (ADI-900-211-0001, ADI-900-212-0001, Enzo Life Sciences, USA). *Cell viability:* Alamar Blue cell viability reagent (Life Technologies, DAL1025) was used to assess cell viability by addition of the 10X, ready-to-use solution to untreated or rapamycin-treated MSCs with and without Mtb infection. Samples were then subjected to fluorometer readings, per the manufacturer’s protocol.

### NO assays

#### Oxidant assay using fluorescent probes

BM-MSCs or human THP-1 macrophages were plated in 96-well plates at a density of 2 × 10^3^ cells per well in triplicate wells and treated with Mtb (MOI 1:5). Cells were then treated with fluorescent probes for the quantification of NO using diaminofluorescein diacetate (DAF-2 DA), per the manufacturer’s instructions (Enzo Life Sciences, USA). Likewise, DCFDA was used to detect ROS. Quantification of NO release was conducted by reading the fluorescence signal over time for uninfected and infected MSCs vs. THP-1 macrophages at ex. 485 nm/em. 515 nm and analyzed by plotting AFUs ( ± SD) against time using Ascent Software version 2.6. *CFU assay after NO inhibition*: MSCs were incubated in the presence of L-NMMA (100 µM) and infected with Mtb for 4 hr; after being washed, the cells were once again incubated with L-NMMA to inhibit NO, and the cells were lysed at time intervals for Mtb CFU counts using 7H11 agar.

### Statistical Analysis

Statistical analyses were conducted using GraphPad 5.0 software. Student’s *t*-test was used for comparisons. Data are expressed as the means ± SD; *p < 0.05, **p < 0.001, ***p < 0.0001. Student’s *t*-test and standard one-way ANOVA followed by Dunnett’s multiple comparison test were used to determine statistical significance. Experiments were repeated a minimum of three times, and at least 2 duplicates were used.

### Ethics statement

All methods were carried out in accordance with relevant guidelines and regulations from Institutional Review board of university of Texas-Houston. All experimental protocols were approved by University of Texas Institutional Biosafety Committee (UT-HSC) that approved the study (HSC-MS-15-0548). Healthy donor-derived bone marrow was from commercial sources and deidentified subjects, and thus no informed consent was necessary for their use in *in vitro* cell culture studies, per the institutional human subjects review committee.

## Electronic supplementary material

supplementary data
