# Supplementary material for: Mesenchymal stem cells internalize Mycobacterium tuberculosis through scavenger receptors and restrict bacterial growth through autophagy
Source: Sci Rep. 2017 Nov 8;7:15010. doi: 10.1038/s41598-017-15290-z (PMC5678154; doi:10.1038/s41598-017-15290-z)
Supplement: Supplementary file 1 — supplementary data [file 41598_2017_15290_MOESM1_ESM.doc]

**SUPPLEMENTAL INFORMATION**

**Mesenchymal stem cells internalize *Mycobacterium tuberculosis* through scavenger receptors and restrict bacterial growth through autophagy**

Arshad Khan, Ph.D.,^1^ Lovepreet Mann, M.D.,^2^ Ramesha Papanna, M.D,^2^ Lyu, Mi-Ae,^2^ Christopher R. Singh, Ph.D.,^1^ Scott Olson, Ph.D.,^3^ N. Tony Eissa, M.D.,^4^ Jeffrey Cirillo, Ph.D.,^5^ Gobardhan Das, Ph.D.,^6^  Robert L. Hunter, M.D. Ph.D., ^1^ and Chinnaswamy Jagannath, Ph.D. ^1^

^1^ Dept. of Pathology and Laboratory Medicine, University of Texas Health Sciences Center, Houston, TX 77030 ^2^ Dept. of Obstetrics, Gynecology and Reproductive Sciences, UTHSC-Houston ^3^ Dept. of Pediatric Surgery, UTHSC-Houston; ^§^

^5^ Dept. of Pulmonary Medicine, Baylor college of Medicine, Houston, TX; ^¶^

^5^ Dept. of Microbial Pathogenesis and Immunology, Center for Airborne Pathogens Research and Imaging, Texas A&M Health Science Center, College of Medicine;

^6^ Center for Molecular Medicine, Jawaharlal Nehru University, New Delhi India.

*“The authors have declared that no conflict of interest exists.”*

*Correspondence to:*

Chinnaswamy Jagannath. Ph. D.

Professor, MSB 2.200, Dept. of Pathology and Laboratory Medicine

University of Texas Health Sciences Center, Houston TX 77030

Email: [Chinnaswamy.Jagannath@uth.tmc.edu](mailto:Chinnaswamy.Jagannath@uth.tmc.edu); Phone: 713 500 5353 Fax: 713 500 0730

**Figure-S1:** Mesenchymal stem cells from human (h) bone marrow, C57Bl/6 mouse derived (M) macrophages and DCs and PMA activated human THP-1 macrophages. Phagocytosis of *M. bovis* BCG is comparable among all four types of phagocytes.


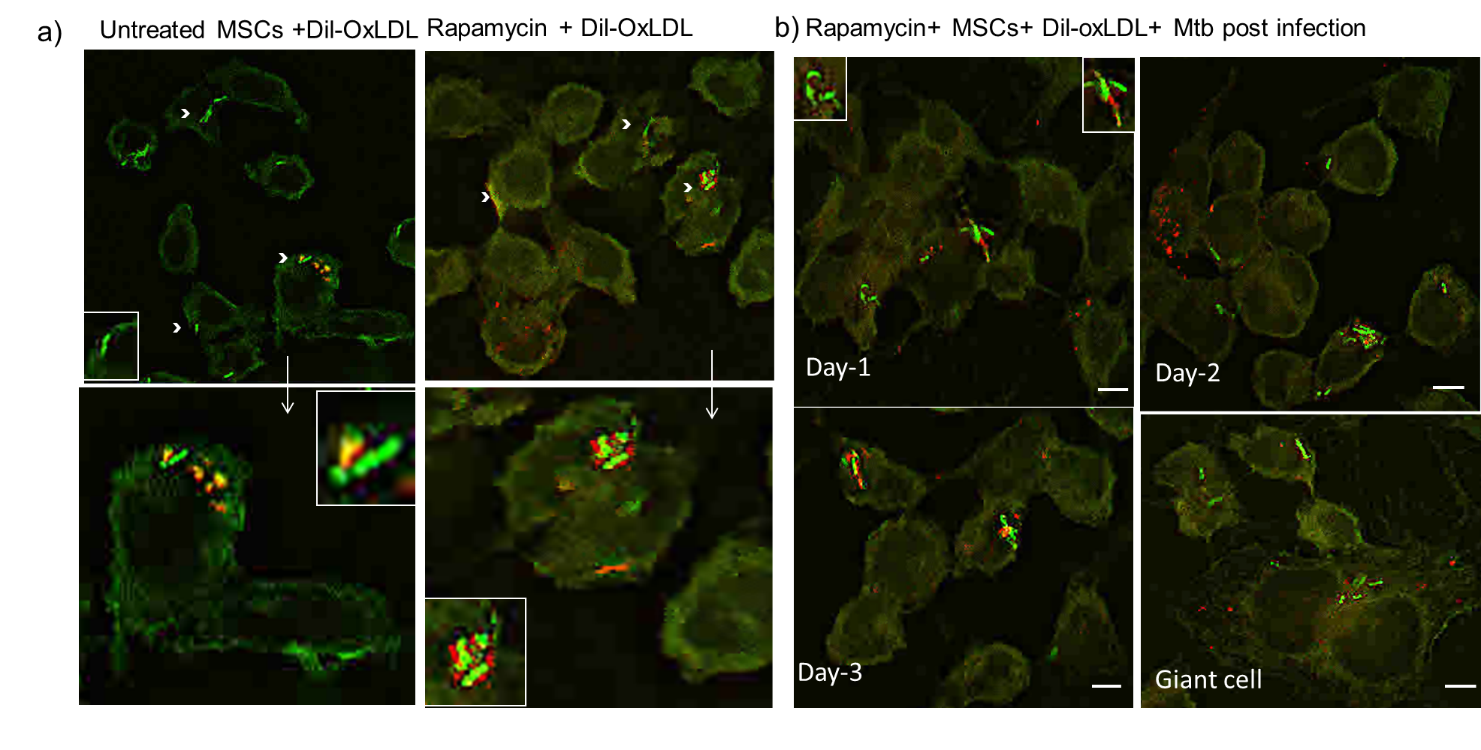
**Figure-S2: Time dependent colocalization of Dil-oxLDL with *rfpMtb* phagosomes in mesenchymal stem cells.** MSCs from bone marrow (BM-MSCs) were incubated with Dil-oxLDL for 90 min, washed and infected with gfpMtb for 4 hr (MOI=1). Washed cells were incubated with or without 5 µM rapamycin at 37^o^C and 5% CO_2_. At 4 hr (a) and intervals (b) were fixed and examined using deconvolution microscopy to quantitate (methods) gfpMtb (green) phagosomes colocalizing with Dil-oxLDL (red) (white bar= 5 µM). Representative panels illustrate colocalization of gfpMtb and Dil-oxLDL over days of incubation. Some MSCs tend to form giant cells by day 3-5.


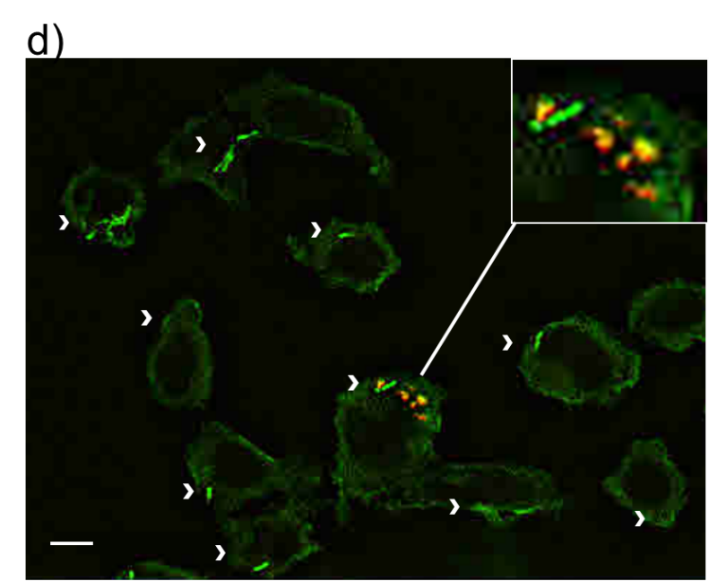
(d) Image in panel-a is expanded. Early after infection at 4 hr, nearly all MSCs cells have at least one CFU of *gfp*Mtb (arrowheads) & each cell contained between 1-5 CFUs. Inset shows Dil-oxLDL colocalizing with gfpMtb.


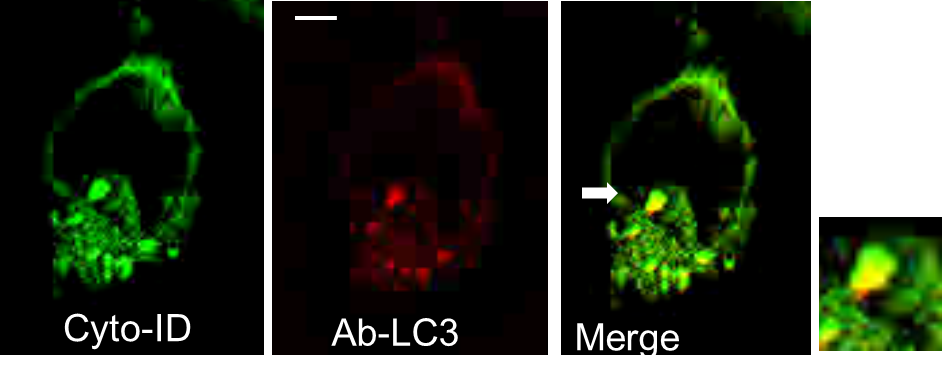
**Figure.S3*:*** BM-MSCs were stained using cyto-ID, fixed with 2.7% paraformadehyde and stained using an antibody to human LC3 and Texas red conjugated anti-human IgG. Both cyto-ID and LC3 antbodies stain the autophagic vesicles (merge, inset).

**
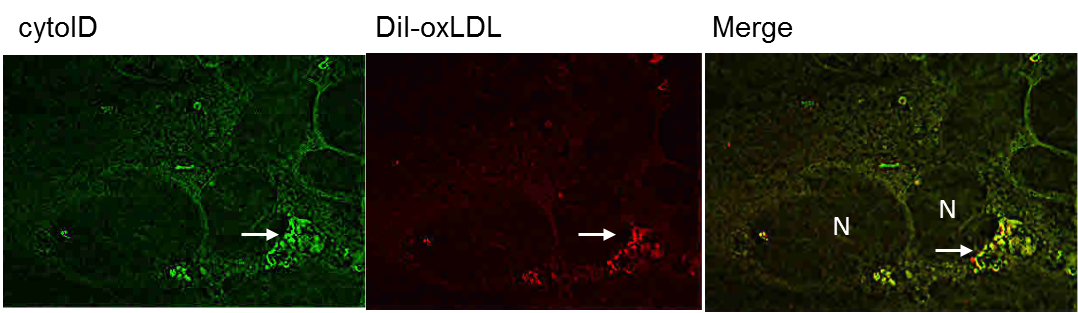
**

**Figure-S4: Colocalization of Dil-oxLDL with *auto*phagosomes in mesenchymal stem cells.** Right: MSCs were incubated with Dil-oxLDL for 90 min, washed and counterstained with cyto-ID. Images analyzed using a deconvolution microscope. (a) Arrow show that endosomes containing Dil-oxLDL colocalize (arrows) with autophagosomes stained with cyto-ID (N=nucleus) Left: Quantitation of autophagic puncta colocalizing with Dil-oxLDL is shown for MSCs pre- and post-rapamycin (1 µM) activation. Two hundred puncta per microscopic field in triplicate chambers of MSCs were counted for colocalization for rapamycin treatment or none at the indicated time points, followed by their averaging and expressed as percent colocalization (SD).

**Figure-S5: *M. tuberculosis grows over time in human macrophages, mouse macrophages and DCs.*** Human THP-1 macrophages were treated with PMA and mouse DCs and macrophages bead purified from bone marrow of C57Bl/6 mice were infected with Mtb using an MOI of 1. Triplicate wells of 10*6 cells per time point were washed and incubated at 37^o^C for 7 days and at intervals cells lysed using 0.01%

**Figure-S6:** (a) *Rapamycin-induced autophagy kills M. tuberculosis strains uniformly within human THP1 macrophages.* Human THP-1 macrophages were plated at 10*6 cells in triplicates and either tested naïve, treated with 3-methyladenine (3MA) or activated overnight with 1 µM of rapamycin, followed by addition of various Mtb strains (MOI=1) as indicated for 4 hr with mixing. Cells were then washed and plated. They were harvested on day 3 (left) or day 1(right) for CFU counts of whole cell lysates on 7H11 agar. (a) Rapamycin activation induces autophagy in THP-1 macrophages and increased killing of intracellular Mtb (p* < 0.008; ANOVA). (Right) 3MA mediated inhibition of autophagy in THP-1 is also illustrated to demonstrate the specificity of the autophagy mediated killing (p* < 0.01; ANOVA). To avoid potential cytotoxicity due to 3-MA, cells were harvested 24 after rapamycin activation and plated. The three single drug resistant strains of Mtb are from ATCC and derived Mtb H37Rv.

(b) To determine whether Rapamycin pretreatment affects uptake of Mtb, BM-MSCs were incubated with 1 and 5 µM of rapamycin followed by infection with Mtb for 4hr. MSCs were washed fixed and intracellular Mtb determined as in Fig.1. Rapamycin had no effect on uptake of Mtb. Similarly, rapamycin had not effect on initial uptake of Mtb in mouse macrophages.

**Figure-S7: Activation of autophagy through rapamycin in mesenchymal stem cells enhances killing of intracellular *M.bovis BCG***. a) BM- MSCs were purified using magnetic beads and either tested naïve or activated with varying doses of rapamycin followed by phagocytosis with *M. bovis* BCG for 4 hr (MOI=1). Washed cells were incubated at 37^o^C and 5% CO_2_ and on times points indicated, cell lysates plated for viable colony (CFU) counts in 7H11 agar. Rapamycin activated MSCs reduce the viability of *M. bovis* BCG by day 5 of incubation (p values by one way ANOVA, 2 experiments). Color panels to the right indicate colocalization of autophagic puncta with rfpBCG organisms.


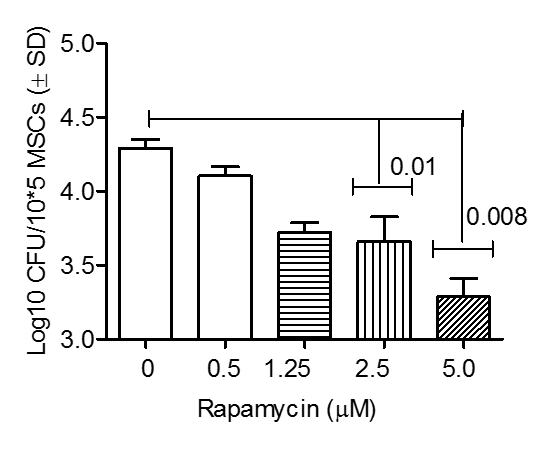

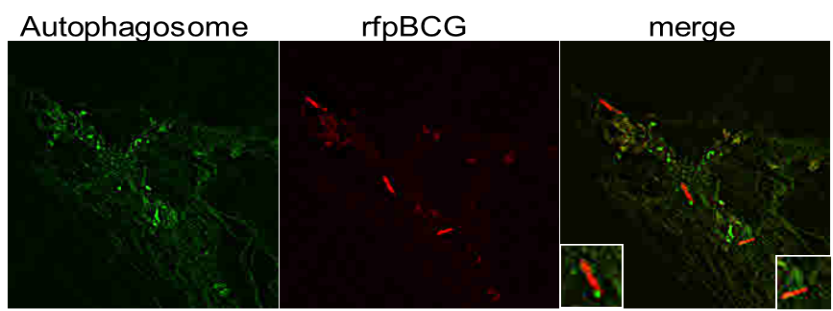


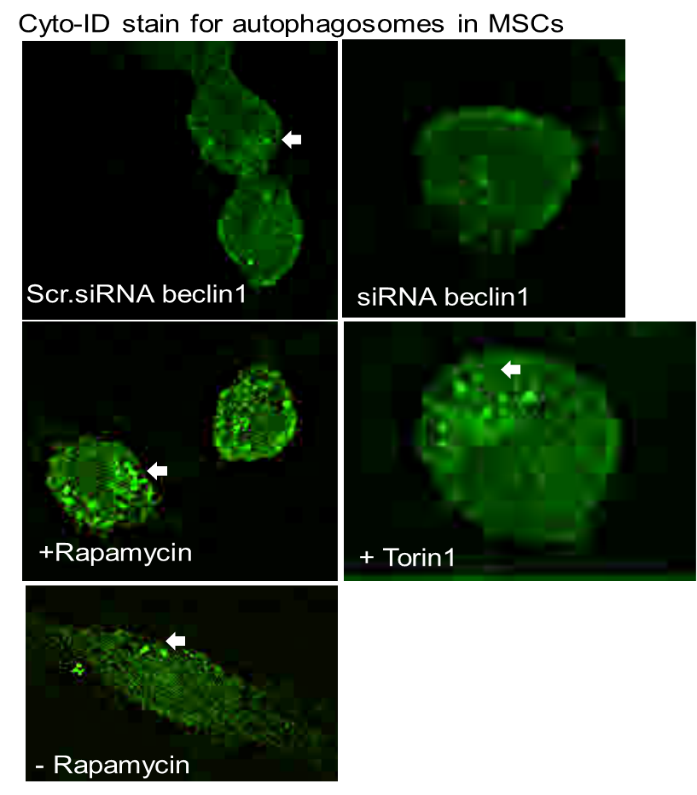
**Figure-S8:** (a) *Autophagosome detection using cyto-ID stain:* (left) Cyto-ID (Enzo) is an amphiphilic tracer dye that preferably labels autophagosomes in macrophages. Representative images of MSCs stained with cyto-D are illustrated in naïve or treated macrophages using deconvolution microscopy.

*(b)Decline of substrates during autophagy.* Intracellular levels of autophagy substrates p62 (sequestosome-1; SQSTM1) and NBR1 in siRNA vs.beclin-1 and scrambled siRNA treated human BM-MSCs with or without rapamycin treatment. MSCs lysates of different treatment groups were collected on day 3 post infection, from triplicate wells, and were estimated for p62 and NBR1 substrates of autophagy using sandwich ELISA (** p< 0.005,* p<0.05, ANOVA). APH indicates up-or down- regulation of autophagy. Western blot analysis confirms the decline in cytosolic beclin1 after siRNA knockdown.

*Raw Images of western blot Fig.7C*

*
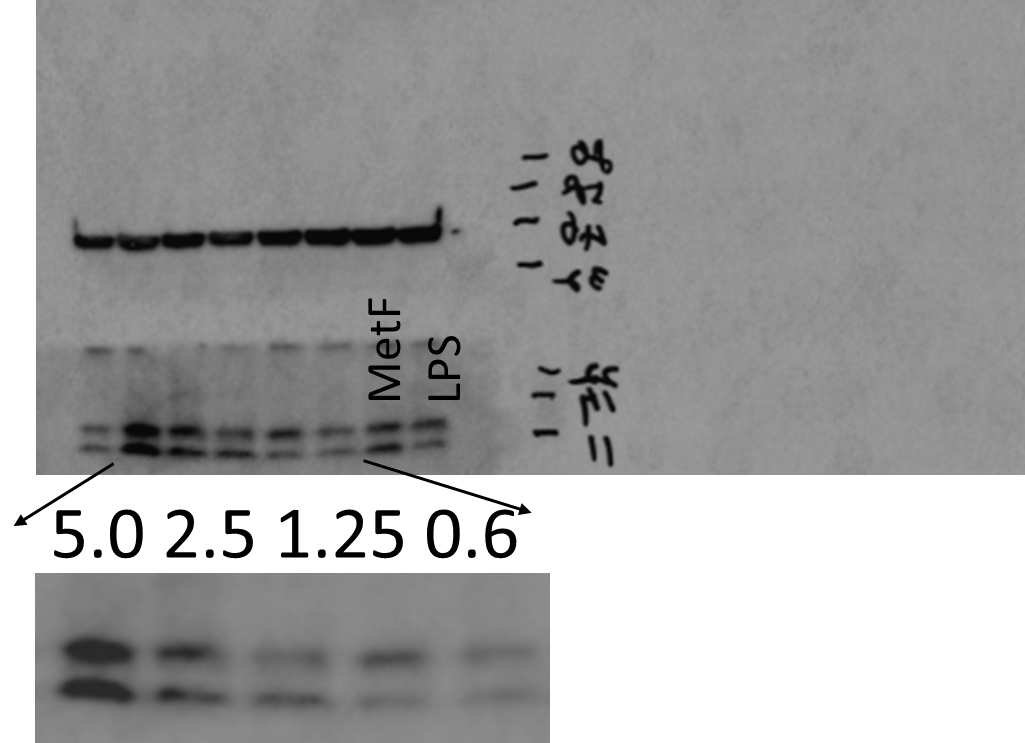
*
